# Semi-Automated Data Analysis for Ion-Selective Electrodes and Arrays Using the R Package ISEtools

**DOI:** 10.3390/s19204544

**Published:** 2019-10-19

**Authors:** Peter W. Dillingham, Basim S.O. Alsaedi, Aleksandar Radu, Christina M. McGraw

**Affiliations:** 1 Department of Mathematics and Statistics, University of Otago, Dunedin 9054, New Zealand; 2 School of Science and Technology, University of New England, Armidale, NSW 2351, Australia; balsaedi@myune.edu.au or; 3 Department of Statistics, University of Tabuk, Tabuk 71491, Saudi Arabia; 4 Lennard-Jones Laboratories, Birchall Centre, Keele University, Keele, Staffordshire ST5 5BG, UK; a.radu@keele.ac.uk; 5 Department of Chemistry, University of Otago, Dunedin 9054, New Zealand

**Keywords:** analytical methods, Bayesian methods, calibration, electrochemistry, limit of detection

## Abstract

A new software package, **ISEtools**, is introduced for use within the popular open-source programming language R that allows Bayesian statistical data analysis techniques to be implemented in a straightforward manner. Incorporating all collected data simultaneously, this Bayesian approach naturally accommodates sensor arrays and provides improved limit of detection estimates, including providing appropriate uncertainty estimates. Utilising >1500 lines of code, **ISEtools** provides a set of three core functions—loadISEdata, describeISE, and analyseISE— for analysing ion-selective electrode data using the Nikolskii–Eisenman equation. The functions call, fit, and extract results from Bayesian models, automatically determining data structures, applying appropriate models, and returning results in an easily interpretable manner and with publication-ready figures. Importantly, while advanced statistical and computationally intensive methods are employed, the functions are designed to be accessible to non-specialists. Here we describe basic features of the package, demonstrated through a worked environmental application.

## 1. Introduction

The R programming language [1] has transformed modern data analysis, allowing free access to its core features and thousands of contributed packages. Here, we introduce **ISEtools** [2], a new package for the statistical analysis of data from ion-selective electrodes (ISEs) available on the Comprehensive R Archive Network (CRAN) [1] and easily installed within R. We also note notation, where R **packages** are in boldface, while R functions or commands are in Courier New font. **ISEtools** implements Bayesian models described by Dillingham et al. [3] that allow analyses to be conducted with single ISEs or jointly using data from sensor arrays. Its intent is to be a resource for the ISE community enabling statistical best practices, with new features added over time according to community demands and needs.

Advances in our understanding of the mechanisms of ISE response has led to increased sensitivity, miniaturisation, and simplified experimental protocols. This has enabled their application in challenging areas, including environmental analysis, wearable sensors, and medical applications [4,5,6,7]. These demanding applications require more sophisticated data analysis techniques, which can accommodate both the multivariate data that arises from sensor arrays and the underlying non-linear response of ISEs. **ISEtools** implements advanced techniques in a straightforward manner, making these analyses accessible to researchers and practitioners alike.

Currently, there is a wide range of reporting practices and methodologies employed by the ISE community, often related to the statistical and computational knowledge of researchers. While best practice should include reporting point estimates of parameters and estimates of their uncertainty [8], it is common in the ISE community to only provide point estimates of important parameters such as slopes, analyte activities, or limits of detection (LOD). Moreover, uncertainty estimates should be expressed through confidence or credible intervals, rather than simply as standard errors, to incorporate the uncertainty due to sample size or the asymmetric shapes of confidence intervals for some ISE parameters. The statistical skills required to implement these best practices depends on the parameter being estimated and the data collected. Examples of advances in estimation include non-linear regression [9], Bayesian techniques that simultaneously utilise measurements from two different ISEs for source separation [10] or arrays of redundant sensors to improve precision [3], and neural networks that incorporate environmental patterns [11].

These statistical approaches add value by synthesising all available data. For example, consider the relatively simple case of estimating the slope of the Nernstian portion of the ISE response curve using linear versus non-linear regression. If using linear regression, uncertainty in the slope is linked to a *t*-distribution with *n* – 2 degrees of freedom (df) for the linear case, or *n* – 3 df for the nonlinear case. However, *n* in linear regression is restricted to calibration data in the Nernstian region, while *n* in non-linear regression is based on all calibration data. This means that a seven-point calibration with three points in the linear range would have a *t*-based multiplier of *t*_1_ = 12.7 for a 95% confidence interval if using linear regression, but *t*_6_ = 2.4 for non-linear regression. That is, when employing linear approximations that use only a subset of the data collected, information is lost and uncertainty increases. Often, a follow-on result is that uncertainty is neither estimated nor reported. Similar issues arise when estimating the activities of experimental samples, compounded by asymmetric sampling distributions in some regions of the response curve or when using standard addition techniques. Similarly, if defined in a probabilistic manner in accordance with IUPAC recommendations [12], the LOD is a highly non-linear function of three parameters resulting in a skewed distribution that may have substantial uncertainty [13].

The goal of **ISEtools** is to make implementing best practices [8,12,14] as simple as possible, for as wide a range of data as possible, and for as many researchers as possible. The version introduced here (Version 3.1.1) implements statistical methods described by [3,13] for single ISEs or ISE arrays of redundant sensors, allowing the estimation of model parameters, experimental activities, and LODs. Additional functionality will be introduced in future versions (e.g., current projects include developing methods to accommodate sensor arrays measuring multiple analytes, estimating LOD for an entire sensor array, and improving statistical methods for estimating selectivity coefficients).

Substantial automation means that researchers can simply load data stored in a text file (e.g., typically from a spreadsheet) using the command loadISEdata. Once loaded, the ISE(s) can be characterised using the command describeISE. If the data includes experimental samples, activities can be estimated using the command analyseISE. In the background, the software package determines the structure of the data (e.g., the number of ISEs or whether standard addition was used, which Bayesian model is appropriate for analysis, and the initial values for numerical procedures). It also calls specialist software to implement the model and processes results for easy interpretability and clear graphical representation.

## 2. Materials and Methods

This section describes the basic statistical model used, data structures supported, a computational overview, and implementation of an analysis of lead in soil. Technical details, definitions, and numerous options are not presented. Instead, readers are referred to the vignette for a detailed description of the package and help files for individual functions. The vignette (ISEtools.pdf) is available from https://CRAN.R-project.org/package=ISEtools, and is also accessible after installing R and loading the **ISEtools** library. Individual help files (e.g., for loadISEdata) are also available once the **ISEtools** library is loaded. These are accessed via:

library(ISEtools); vignette("ISEtools"); help(loadISEdata)

### 2.1. ISE Response Model

**Figure 1:**
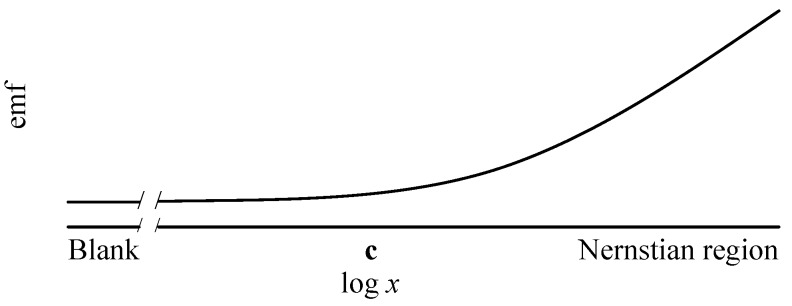
Ion-selective electrode (ISE) response as parameterized in **ISEtools**. The curvilinear portion of the response curve is determined by **c**, while the slope in the Nernstian region is **b** (mV/decade).

Ion-selective electrodes convert analyte activity to an electrical signal [15], with the response described by the empirical version of the Nikolskii–Eisenman equation [3,16] and parameterised as:

(1) y=a+blog(x+c)+error,

where *y* is the electromotive force (emf) response of the ISE; *x* is the activity of the ion of interest; **a** is a baseline emf; **b** is the slope, whose theoretical value is determined by the valence of the primary ion, temperature, and natural constants; **c** is a parameter linked to the interfering ions within the chemical matrix and the selectivity of the ISE to those ions; and the random emf noise follows a normal distribution (i.e., error ~ normal(0, sigma)). Figure 1 shows the expected response of a single ISE, including the flat region (when activity *x* << **c**) that cannot reliably be distinguished from a blank, and the Nernstian region (when *x* >> **c**) in which linear regression methods may be usefully employed. **ISEtools** was specifically developed for applications with data across the full response curve, but also works for datasets entirely within the Nernstian region. 

### 2.2. Computational Overview

**ISEtools** implements Bayesian methods described by [3,13], and operates within R [1] or the related RStudio [17], interfacing with an additional programme to run the Bayesian analyses, OpenBUGS [18] or jags [19], both based on the BUGS [20] language. Users with basic familiarity of R or other scripting languages will have an advantage getting started, but will not need familiarity with the Bayesian programmes. Installation requires R or RStudio as well as OpenBUGS or jags, all of which are free, easily accessible through simple web searches, and straightforward to install. We recommend OpenBUGS, but include a jags option for macOS users. Users must also install R packages **ISEtools**, **Xmisc** [21], **coda** [22], and either **R2WinBUGS** [23] and **BRugs** [18] (if using OpenBUGS) or **rjags** [24] (if using jags) through the built-in interface in R or RStudio. **Xmisc** and **coda** are often installed automatically as dependencies of **ISEtools**, depending on user settings in R.

Users use three core functions: loadISEdata to import and process the ISE data, describeISE to characterise ISEs using calibration data (e.g., to estimate model parameters, LODs, and their uncertainty), and analyseISE to estimate unknown activities of experimental samples. In conjunction with each function are print, summary, and plot commands to summarise and visualise model output.

### 2.3. Data Structures and Estimation 

**ISEtools** is designed to work with calibration data, where *x* and *y* are both observed, to estimate model parameters a, b, c, and sigma. This also allows estimation of LOD*_α,β_* based on rates of false positives (*α*) and negatives (*β*) as recommended by IUPAC [12], using defaults of *α* = *β* = 0.05. We note that this is not the commonly used LOD calculation for ISEs [25], which is based on the intersection of the Nernstian line with a blank but does not meet general IUPAC recommendations for LODs.

When combined with experimental data, where *y* is observed but *x* is unknown, inverse methods [3] are used to estimate unknown activities (*x*)*,* conditional on the model parameters estimated from the calibration data. **ISEtools** accommodates experimental data in Basic format, where an emf is recorded for each experimental sample, or in Standard Addition format, where an aliquot with known activity and volume is added to each experimental sample and emf is recorded before and after the addition. 

**Figure 2:**
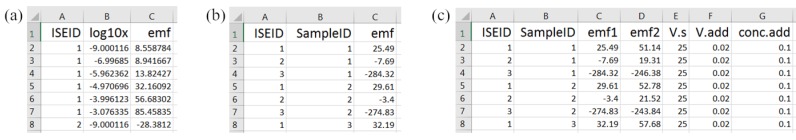
Structure of the (**a**) calibration data and (**b**,**c**) experimental data for three ISEs measuring lead in soil. Typically, users would record experimental data in either (**b**) Basic or (**c**) Standard Addition formats. Both are shown for illustrative purposes.

The structure of the data files for an array of three ISEs is shown for calibration data (Figure 2a), experimental data in the Basic format (Figure 2b), and experimental data in the Standard Addition format (Figure 2c). 

Variable definitions are intuitive and fully described in the **ISEtools** vignette and help files. The calibration data includes variables ISEID (indicating which ISE recorded the data), log10x (the log of the known activity of the calibration samples), and emf (the recorded emf in mV). The experimental data in the Basic format has variables ISEID, emf, and SampleID (indicating which sample is being measured). The experimental data in the Standard Addition format has variables ISEID, SampleID, emf1 (the emf before the aliquot is added), emf2 (the emf after the aliquot is added), V.s (the volume of the original sample), V.add (the volume of the aliquot), and conc.add (the activity of the aliquot).

**ISEtools** employs Bayesian methods rather than alternatives such as non-linear regression via least squares or maximum likelihood. Briefly, any prior knowledge about random variables (e.g., model parameters or analyte activity) is updated by a probability model linking observed data to those parameters. The probability model is based on the Nikolskii–Eisenman equation (Equation (1)), with adaptations for sensor arrays or standard addition data where appropriate. This provides a posterior probability distribution for the random variables via Bayes’ theorem, typically presented as credible intervals that are broadly analogous to confidence intervals. Relative to other ISE data analysis approaches, the key benefit of Bayesian methods is their ability to incorporate all data into a single model and maximise information. Particularly, computational tools such as OpenBUGS and jags allow consistent implementation of both simple and complex models. Further details of the statistical models are available in the Supporting Information of [3], particularly Equations S-1 through S-3, with OpenBUGS code shown in Figures S-2 through S-4.

For the simplest case of characterising ISE parameters using calibration data, Bayesian methods produce point estimates and uncertainty intervals very similar to those from non-linear regression. Similarly, if only one ISE is used and experimental data are in Basic format, inversion of a prediction interval from non-linear regression produces similar estimates and intervals. If all data are in the Nernstian region, these both simplify to linear regression results. However, in other contexts the Bayesian approach has clear advantages including supporting complex sampling distributions, non-standard data sources, and multivariate data from sensor arrays [3,10,13].

These advantages revolve around: (1) LOD estimation, where the ability to sample from the joint posterior distribution of model parameters allows straightforward calculation of its distribution; (2) estimation of activity when standard addition is used and the sampling distribution may be highly asymmetric; and (3) estimation of activity when multiple sensors are used in an array. For this final case, the Bayesian treatment of unknown activity *x* as a random variable allows all available data (e.g., multiple ISEs of varying quality measuring the same sample) to be used simultaneously to find the posterior distribution for *x* and calculate its 95% credible interval. Further, the model appropriately weighs data based on individual sensor quality (i.e., noisy sensors are automatically down-weighted). **ISEtools** currently accommodates sensor arrays of the same type of ISE, and future versions will accommodate arrays of different ISEs.

### 2.4. Analysis of Lead in Soil

**Figure 3:**
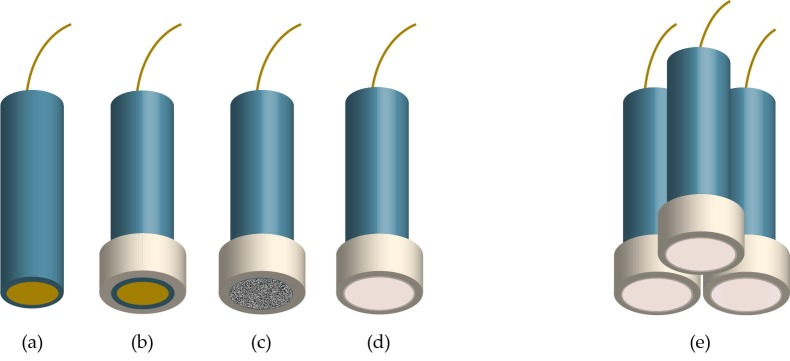
Each of the Pb^2+^ solid-contact ISEs were prepared from gold-coated copper rods in a sheath of Teflon tubing (**a**). PVC tubing was then added to provide a protective sleeve for the sensing face (**b**). Next, a conducting polymer layer was drop cast onto the gold surface (**c**), before drop casting the Pb^2+^-selective membrane (**d**). Finally, three Pb^2+^-selective membranes were combined into a three-ISE array (**e**).

Next, we show a worked example using **ISEtools** from an array of three solid-contact ISEs measuring lead in soil. The purpose of this example is to demonstrate the implementation and relative ease of analysis using **ISEtools**. Manufacturing methods for the ISE array have been described previously [26]. Briefly, the Pb^2+^-selective membrane was prepared by dissolving 5 mmol kg^−1^ sodium tetrakis[3,5-bis(trifluoromethyl)] phenylborate, 12 mmol kg^−1^ lead ionophore IV, 32 wt% poly(vinyl chloride) (PVC) and 66 wt% of bis(2-ethylhexyl)sebacate in tetrahydrofuran. This composition was based on a membrane developed for trace-level measurement of lead in rivers, lakes, and tap water [27]. Three solid-contact electrodes were prepared from Teflon-coated copper rods (3 mm diameter). The face of the sensing end was polished and sputter-coated with gold before adding a protective sleeve of PVC tubing. The gold was then coated with a drop-cast layer of a polyoctylthiophene conducting polymer before drop casting the Pb^2+^-selective membrane onto the conducting polymer layer (Figure 3).

Soil samples, collected at abandoned mining sites near Silvermines, County Tipperary, Ireland, were dried and ground before extraction of exchangeable metals through sonication in 1.0 × 10^−3^ M HNO_3_. A four-channel, high-input impedance data acquisition system (World Precision Instruments, Sarasota, FL) was used to simultaneously measure the differences in potential between the three ISEs and a silver/silver chloride reference electrode. After the ISE array was calibrated, Pb^2+^ activity in each sample was estimated using a standard addition approach. All emf values were corrected for liquid-junction potential using the Henderson equation, and ion activities were calculated according to the Debye–Hückel approximation. Here, we used the calibration data and standard addition data from the 17 soil samples to estimate the Pb^2+^ activity and characterise the three ISEs.

Data were first stored in an Excel file but subsequently saved as tab-delimited text files in the format expected by **ISEtools** and are included with the **ISEtools** package in the “/extdata” sub-folder of the **ISEtools** library (e.g., <pathname to R libraries>/ISEtools/extdata). In the example below, the pathname was “C:/Program Files/R/R-3.5.2/library/”. In addition to calibration data (Lead_calibration.txt), the experimental data are available in the Basic (Lead_experimentalBasic.txt) and Standard Addition (Lead_experimentalSA.txt) formats (Figure 2).

## 3. Results

Computational details, syntax, and analysis results for the analysis discussed previously are described below, demonstrating the three key functions of **ISEtools**.

### 3.1. Loading Lead Data

After installing all required software and R packages, the **ISEtools** library was called and the lead data was loaded using loadISEdata. This simply required specifying the locations of the calibration and experimental data files:

library(ISEtools)

lead.example = loadISEdata(

filename.calibration =

“C:/Program Files/R/R-3.5.2/library/

ISEtools/extdata/Lead_calibration.txt”,

filename.experimental =

"C:/Program Files/R/R-3.5.2/library/

ISEtools/extdata/Lead_experimentalSA.txt")

The loadISEdata function imports and processes the data, determining that multiple ISEs were used, and experimental data were present in Standard Addition format. Data can be further examined using print(lead.example) or plot(lead.example) to ensure there are no data entry errors or unusual datapoints, where print and plot functions have been customised for ISEdata objects.

### 3.2. Characterising the ISEs

Once satisfied with the data quality, ISE model parameters are estimated via describeISE. The describeISE function takes data loaded using loadISEdata, the valence (Z) of the primary ion (here, 2 for Pb^2+^) and (optionally, if much different from room temperature) the temperature:

lead.analysis1 = describeISE(lead.example, Z=2, temperature=21)

Because loadISEdata pre-processes the text files, calibration data and the number of ISEs are automatically passed to describeISE. This allows describeISE to automatically apply the appropriate Bayesian model when it calls OpenBUGS or jags; users also have the option to specify their own model. At this point, OpenBUGS or jags implements Markov chain Monte Carlo methods, returning results to R and saved as lead.analysis1. Parameter estimates and the LOD, as well as lower and upper values from 95% credible intervals, are displayed using the print command, again customised to print relevant output for each ISE. For ISE #1, print(lead.analysis1) produces:

Non-linear parameter estimates and 95% CIs for

y = a + b log(x + c)

ISE #1:

Parameter estimate Lower limit Upper limit

a   1.76e+02      1.60e+02    1.93e+02

b   2.95e+01      2.56e+01    3.43e+01

c   2.18e-06      1.03e-06    4.66e-06

sigma 1.42e+00      6.12e-01    5.94e+00

Estimated log LOD{alpha=0.05, beta=0.05} (95% CI): -6.03 (-6.44, -5.03)

**Figure 4:**
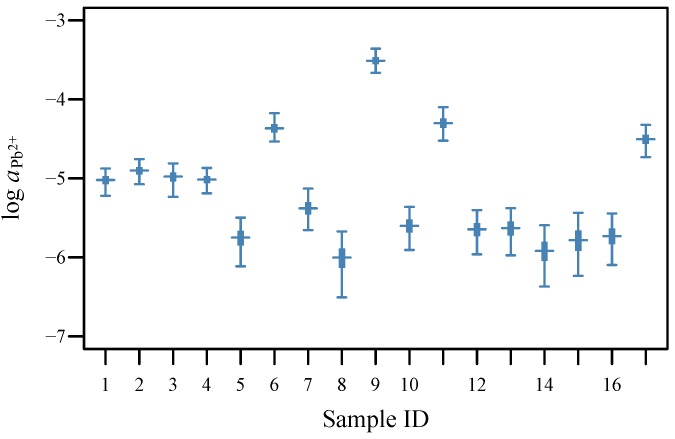
Analysis of 17 experimental soil samples from Silvermines, Ireland using an array of three solid-contact ISEs with Standard Addition data. The 95% (thin lines) and 50% (thick lines) credible intervals and point estimates (–) are shown. The analysis and plot were produced using the R package **ISEtools**.

From this, we see that ISE #1 had close to the ideal Nernstian slope, estimated as **b** = 29.5 mV/decade (95% CI 25.6–34.3 mV/decade), with an LOD near 10^-6^. This information can be used when developing new ISEs and when testing whether they are fit for purpose [13]. For example, lead activity for some of the experimental soil samples (Figure 4) was near this LOD, indicating that, by itself, ISE #1 would struggle to distinguish the lower activities at this site from a blank. If the full distribution of the parameters was of interest, plot(lead.analysis1) would be used instead.

### 3.3. Estimating Activity of Experimental Samples

To estimate analyte activity in experimental samples, analyseISE was used. Here, we used the three ISEs to analyse the 17 experimental soil samples measured using standard addition:

lead.analysis2 = analyseISE(lead.example, Z=2, temperature=21).

As with describeISE, the structure of the data informs the Bayesian model to use with analyseISE and engages OpenBUGS or jags. Again, print and plot commands can be applied to the results. Here, we plot the estimated activities, combining data from three ISEs (Figure 4), and include options to produce appropriate axis labels, specify the plot range, and set the colour using standard R options:

plot(lead.analysis2,

ylab=expression(paste("log ", italic(a)[Pb^{paste("2","+")}])),

ylim=c(-7, -3), col="steelblue")

## 4. Discussion

One of the key benefits of the **ISEtools** package is that it provides access to advanced statistical models for the non-specialist. These models utilise all data (i.e., data beyond just the linear portion of the response) to provide improved activity estimates, particularly in the context of sensor arrays or standard addition data. They also estimate the limit of detection following recommendations by IUPAC and provide uncertainty for those estimates.

This manuscript introduced the **ISEtools** package. Additional detail, including information on data formatting, numerical details, and advanced options, are described in the **ISEtools** vignette. We recommend that interested researchers read the vignette prior to running any analyses. For researchers and practitioners experienced with R, implementation of **ISEtools** should be straightforward. For those without experience, there is an R learning curve to overcome. Fortunately, with millions of users and its open-source nature, there are many “getting started” tutorials available on the web, along with numerous books. We note that many new users prefer RStudio to the base version of R.

Finally, we reiterate that this is a developing package, and look to the ISE community for feedback and future development needs.
